# Megakaryocytes form linear podosomes devoid of digestive properties to remodel medullar matrix

**DOI:** 10.1038/s41598-022-10215-x

**Published:** 2022-04-15

**Authors:** Antoine Oprescu, Déborah Michel, Adrien Antkowiak, Elodie Vega, Julien Viaud, Sara A. Courtneidge, Anita Eckly, Henri de la Salle, Gaëtan Chicanne, Catherine Léon, Bernard Payrastre, Frédérique Gaits-Iacovoni

**Affiliations:** 1 INSERM, UMR1297, Université Toulouse III, Institut des Maladies Métaboliques et Cardiovasculaires, Toulouse, France; 2 Department of Cell, Development and Cancer Biology, Oregon Health & Science University, Oregon, USA; 3 INSERM, UMR_S1255, Université de Strasbourg, Etablissement Français du Sang-GEST, Strasbourg, France; 4 CHU de Toulouse, laboratoire d’Hématologie, Toulouse, France; 5 Molecular, Cellular and Developmental Biology Department (MCD, UMR5077), Centre de Biologie Intégrative (CBI, FR3743), University of Toulouse, CNRS, UPS, 31062 Toulouse, France

**Keywords:** Cell biology, Cell migration, Cellular imaging, Cytoskeleton

## Abstract

Bone marrow megakaryocytes (MKs) undergo a maturation involving contacts with the microenvironment before extending proplatelets through sinusoids to deliver platelets in the bloodstream. We demonstrated that MKs assemble linear F-actin-enriched podosomes on collagen I fibers. Microscopy analysis evidenced an inverse correlation between the number of dot-like versus linear podosomes over time. Confocal videomicroscopy confirmed that they derived from each-other. This dynamics was dependent on myosin IIA. Importantly, MKs progenitors expressed the Tks4/5 adaptors, displayed a strong gelatinolytic ability and did not form linear podosomes. While maturing, MKs lost Tks expression together with digestive ability. However, those MKs were still able to remodel the matrix by exerting traction on collagen I fibers through a collaboration between GPVI, ß1 integrin and linear podosomes. Our data demonstrated that a change in structure and composition of podosomes accounted for the shift of function during megakaryopoiesis. These data highlight the fact that members of the invadosome family could correspond to different maturation status of the same entity, to adapt to functional responses required by differentiation stages of the cell that bears them.

## Introduction

Megakaryocyte (MKs) maturation within the bone marrow and release of proplatelet (PPTs) extensions into the blood flow is essential for platelet production^1^. This complex process is tightly regulated by local cytokines and contacts with the extracellular matrix (ECM)^2,3^. While this notion has been challenged^4^, it is nevertheless clear that MKs maturation starts from progenitors (P) in the osteoblastic niche, enriched in stiff bone matrix and fibronectin, and ends in the vascular niche, near the sinusoids, where collagen IV, laminin and fibrinogen are more abundant^5^. Importantly, collagen I, the most abundant matrix in the body, is also the most represented throughout the bone marrow^6^. MKs were demonstrated to interact with the ECM by forming podosomes, which are F-actin-based structures connecting the ECM to the cytoskeleton to trigger signaling^7^. They belong to the invadosome family that encompasses invadopodia in cancer cells and podosomes in non-transformed cells. Invadosomes are formed by many cells displaying adhesive, motile and invasive properties^8–10^. They also function as mechanosensitive structures, which react to the stiffness of the matrix. Invadosomes are involved in invasion by being preferential sites of metalloproteinase secretion (MMP2 and MMP9) or membrane exposure (MT1-MMP). They display a typical structure formed by an F-actin core including the Arp2/3 complex, src kinase, WASp (Wiskott–Aldrich Syndrome protein), the Cdc42 GTPase and cortactin, which is surrounded by a ring of vinculin, paxillin, talin, mechanosensitive proteins and ECM receptors such as the ß1 integrin^11–14^. Lateral F-actin fibers connect adjacent podosomes and are linked to myosin which is the main regulator of inter-podosomes dymanics^15^. MKs podosomes seem to display sensing activity required for MKs protrusions to detect preferential sites for trans-endothelium crossing, and locally act on basement membranes^16^.

Mutations in genes encoding podosomes proteins often occur in the development of pathologies, from vascular diseases to immune defects or cancer^17,18^. For instance, the mutation of WASp leads to a defect in podosomes formation resulting in defective chemotaxis in macrophages, defective bone resorption in osteoclasts and platelet production by MKs^19^. Podosomes-like WASp-dependent structures refered to as ‘actin-nodules’ were described in platelets where they contribute to aggregation via ECM–platelet and platelet–platelet contacts^20^. This observation prompted us to revisit the role and structure of podosomes in MKs, before the stage of endothelium crossing^16^.

Our findings demonstrated that upon plating on fibrillar collagen I, mature MKs assembled F-actin-based linear structures as well as dot-like podosomes, which composition was similar. We therefore called them 'linear podosomes' by analogy to 'linear invadopodia'. Interestingly, MKs progenitors displayed strong degradative ability, which decreased through maturation in parallel to the loss of Tks5. Videomicroscopy showed for the first time that linear podosomes largely derived from the fusion of interconnected single podosomes aligned along the fibers. Dynamics of individual podosomes in close vicinity along fibers was depending on the activity of myosin IIA. We found that linear podosomes bound collagen I through GPVI and the ß1 integrin. While no digestive activity was present, linear podosomes remodeled the matrix via mechanical traction. When collagen was immobilized by crosslinking, mature MKs reacted by extending PPTs-like protrusions along the fibers. Our data demonstrate that the physiological maturation of bone marrow progenitors into MKs was paralleled by the maturation of digestive Tks5-positive dot-like podosomes into linear-podosomes that remodeled the ECM by mecanotransduction of traction forces. These observations strongly suggest that the different forms and composition of the invadosomes family members could correspond to specific maturation stages/forms required for a specific cell type to properly respond to environmental cues.

## Results

### Mature MKs form linear F-actin structures on fibrillar collagen I

**Figure 1:**
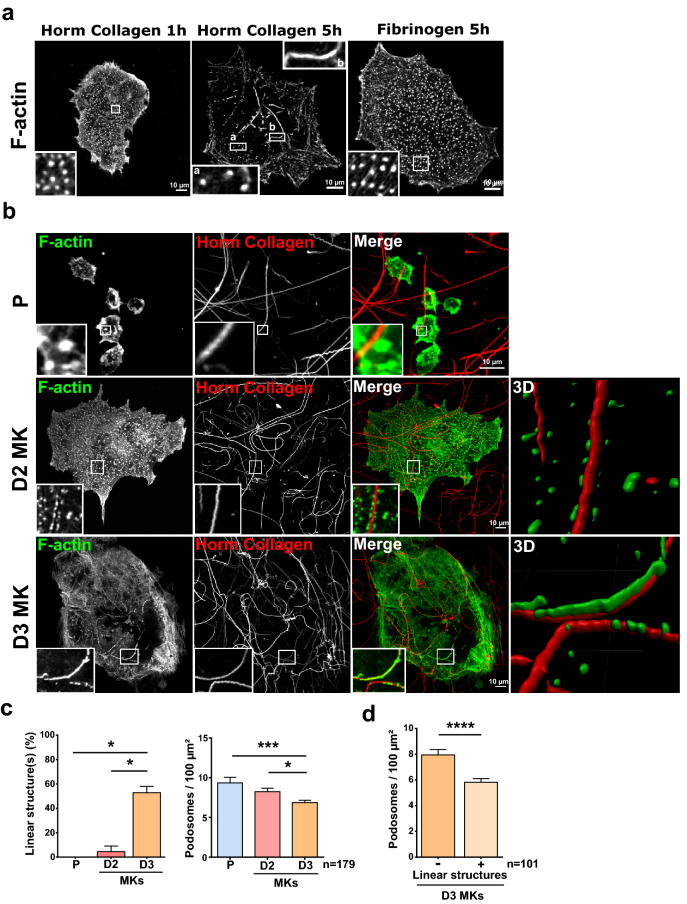
Mature MKs form linear F-actin structures on fibrillar collagen I. (**a**) MKs were spread for 1 h or 5 h on glass surface coated with 100 µg/ml Horm collagen or 100 µg/ml fibrinogen. Cells were then fixed and permeabilized before staining for F-actin. Box a shows dot-like podosomes, box b shows linear actin structure along the collagen I fiber. Scale bars = 10 µm. (**b**) Representative confocal images of Lin^−^ progenitor (P), MKs differentiated for 2 days (D2MK), or 3 days (D3MK) with mTPO (50 ng/ml), cultured on 100 µg/ml labelled-Horm collagen, scale bars = 10 µm. 3D corresponds to a 3D surface rendering of the zoomed areas using the IMARIS software. (**c**) Quantification of the percentage of cells with linear F-actin structures and of dot-like podosomes (podosomes) density according to the indicated maturation time. Values are from 4 independent experiments. (**d**) Quantification of dot-like podosomes (podosomes) density (for 100 µm^2^) among D3MKs according to the presence (+) or not (−) of linear F-actin structures. Values (mean ± S.E.M.) are from 4 independent experiments. *P < 0.05, ***P < 0.001 according to the Kruskall–Wallis test, ****P < 0.0001 according to the Mann–Whitney test. n = number of cells studied.

As already mentioned, MKs were demonstrated to form podosomes on several different matrices within the bone marrow^7^. The properties of podosomes have been documented, but their composition and function remained elusive in MKs. We therefore sought to investigate whether they were similar to the digestive linear-invadosomes/invadopodia described in endothelial, fibroblasts and tumoral cells^7,21,22^, or if they corresponded to a different type of podosomes associated with functions specific to MK particular biology. MKs differentiated for 3 days with mTPO (last maturation stage before proplatelets emission^23^) were first plated onto fibrinogen and fibrillar collagen I (Horm collagen), then the status of F-actin was assessed by confocal microsocopy. As expected, dot-like podosomes were formed on both matrices (Fig. 1a). Interestingly, after 5 h of spreading, MKs plated on fibrillar collagen I clearly displayed linear F-actin bundles along collagen I fibers, while the number of dot-like podosomes decreased. Podosomes remained unchanged on fibrinogen after several hours (Fig. 1a). The Formation of the linear structures was time-dependent and peaked 5 h post-plating. We then investigated whether it correlated with the stage of MKs maturation. Lin^−^ progenitors (P), MKs differentiated in mTPO (50 ng/ml) for 2 days (D2MK) or 3 days (D3MK), were plated on labelled-Horm collagen and F-actin observed after 5 h. Figure 1b demonstrated that progenitors assembled only dot-like podosomes and did not seem to specifically react to collagen I fibers. After 2 days of differentiation, alignment of dot-like podosomes were observed along fibers, suggesting that MKs were acquiring the ability to sense stiff fibrillar collagen I (Fig. 1b 3D surface rendering, Supplementary video 1). Interestingly, only fully mature D3MKs formed F-actin bundles over collagen I (Fig. 1b 3D surface rendering, Supplementary video 2). About 52.9% of D3MKs, versus only 4.5% of D2MKs displayed the linear actin structures, always associated to collagen I (Supplementary Fig. S1a), while dot-like podosomes number decreased (Fig. 1c,d). These data indicated that formation of linear actin bundles on collagen I was dependent on MKs maturation state.

### Dot-like podosomes and linear F-actin associated to collagen I fibers are two forms of the same entity

**Figure 2:**
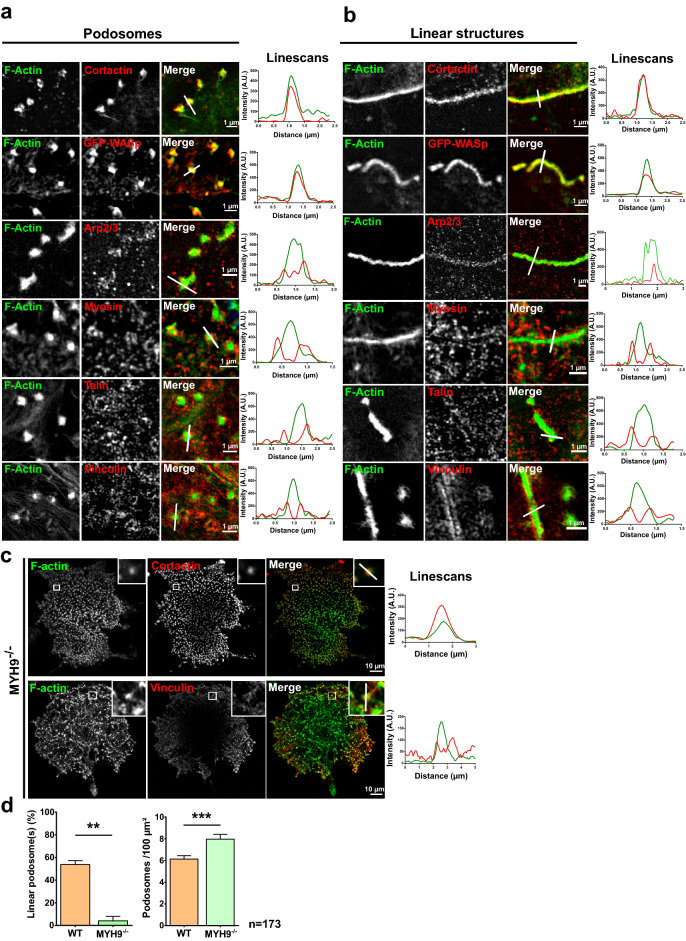
Classical podosomes and collagen-associated F-actin structures share the same protein composition. D3MKs were cultured on 100 µg/ml Horm collagen for 5 h then fixed and permeabilized before staining for F-actin (green) and labeled for cortactin, Arp2/3, myosin IIA, talin and vinculin (red). GFP-WASp was transduced using lentivirus in P and cultured with 50 ng/ml mTPO for 3 days before being processed. Images were taken using SIM. (**a**) Images correspond to dot podosomes (**b**) and linear F-actin structures. Linescans show the distribution of fluorescence along the white line. Scale bars = 1 µm. (**c**) MYH9^−/−^ D3MKs were treated as above before staining for F-actin, cortactin or vinculin. Scale bars = 10 µm. (**d**) Quantification of percentage of WT and MYH9^−/−^ MKs forming linear podosomes and of dot-like podosomes (podosomes) density (for 100 µm^2^). Values (mean ± S.E.M.) are from 5 independent experiments. **P < 0.01 and ***P < 0.001 according to the Mann–Whitney test). n = number of cells studied.

To elucidate whether podosomes and these F-actin structures were related, we analyzed their respective protein composition using super-resolution SIM microscopy (Structured Illumination Microscopy). Dot-like podosomes appear as the well-known F-actin core surrounded by an adhesive ‘ring’, which corresponds more to a cloud of protein clusters when observed by super-resolution^12,15,24,25^. Observations clearly demonstrated that both structures contained similar proteins. Indeed, F-actin, WASp, cortactin and Arp2/3, hallmarks of podosomes, were present in the core, while myosin IIA, talin and vinculin were detected as small clusters within the ring (Fig. 2a). Interestingly, SIM showed without ambiguity that linear actin bundles did not exist as an alignment of intact dot-like podosomes (core and ring clustered together) on the collagen fibers. In MKs, we observed a rearrangement of podosomes components to form a linear podosome in which the core became an elongated structure covering the collagen fiber and containing F-actin, cortactin, WASp and Arp2/3, while surrounded by longitudinal clusters of the proteins from the ring: myosin IIA, talin and vinculin (Fig. 2b), which gave the appearance of a ‘gutter’ around the longitudinal core of the linear podosome (Supplementary Fig. S2a, YZ projection).

Interestingly, all linear podosomes were formed on collagen I fibers, but not all the fibers generated similar linear podosomes. A correlation was observed between the size of the collagen I fibers and the structure of the associated linear podosome, with thick fibers appearing to trigger the formation of gutter-like linear podosomes (Fig. 1b and Supplementary Fig. S2a,b, compare width on line scans of both type of fibers). Supplementary Figure S2c graph demonstrated that gutters formed preferentially when the fibers width was around twice the width of a single podosomes (around 1 µm for the collagen while the diameter of a dot-like podosome in MKs is about 0.5 µm)^25,26^. Hence, fibers thickness appeared important to drive the structuration of linear podosomes.

Importantly, both types of podosomes contained myosin IIA, the main isoform in MKs (Fig. 2a,b). Actomyosin is the main regulator of podosomes dynamics^12,15,27^. MKs from MYH9^−/−^ mices (KO for myosin IIA), were only able to form evenly spread dot-like podosomes (Fig. 2c). Linear podosomes formation was totally abrogated by the lack of myosin IIA (Fig. 2c,d). Pharmacological inhibition of myosin II by 20 µM of blebbistatin led to the same conclusion (Supplementary Fig. S5). Therefore, these data showed that actomyosin contraction was necessary for the formation of linear podosomes in MKs.

### Dynamics fusion and fission of dot-like podosomes drive the formation of linear podosomes

**Figure 3:**
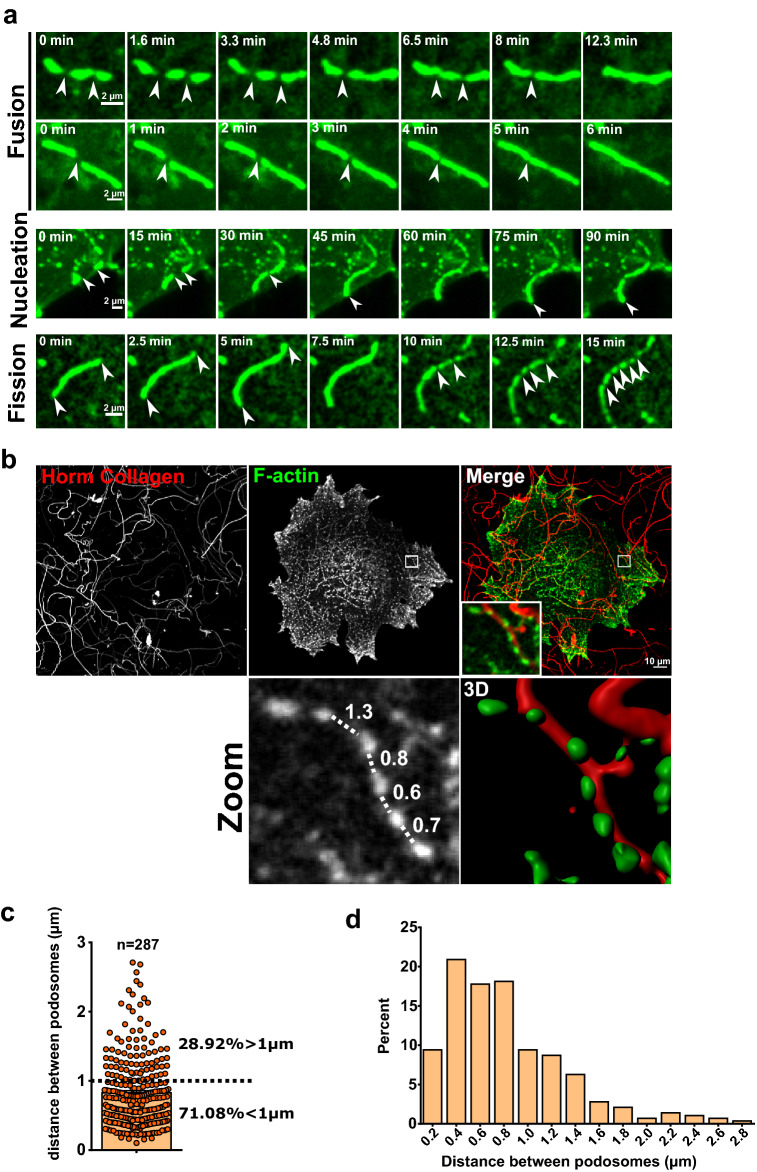
Linear podosomes derive from dynamic fusion of classical dot podosomes. (**a**) Transduced Lifeact-GFP D3MKs were cultured on 100 µg/ml Horm Collagen for 5 h. Movies obtained from confocal videomicroscopy showed podosomes dynamics (fusion, nucleation, fission) at the indicated times. Scale bars = 2 µm. (**b**) Representative images of D3MK cultured on labeled 100 µg/ml Horm Collagen for 5 h. Zooms show respectively the distances (µm) between each dot podosomes along a collagen fiber and the 3D surface rendering of the structure realized with IMARIS. Scale bar = 10 µm. (**c**) Graphical representation of the quantification of distances between dot-like podosomes. Percentages of podosomes separated by more or less of 1 µm are indicated. (**d**) Percentage of the distribution of dot-like podosomes interval along collagen fibers. Data represent analysis of 287 podosomes from D3MKs.

Because the number of linear podosomes increased while dot-like podosomes decreased, we hypothesized that both structures derived from each other upon interaction with fibrillar collagen I. Confocal videomicroscopy of MKs expressing Lifeact-GFP demonstrated that most of the formation of linear podosomes resulted from the fusion of individual dot-like or small linear podosomes (Fig. 3a, white arrow indicates the point of junction). Fusion of pre-existing podosomes appeared to be effective since it was completed in an average of 10–15 min (Supplementary videos 3 and 4). However, linear podosomes also formed by nucleation at the extremity of a pre-existing linear podosome (Fig. 3a: middle panel, fusion then nucleation; lower panel on both extremities). Nucleation, however, seemed to be more time consuming (over 90 min in average), probably because of the difficulty of assembling de novo a complex protein network. Podosomes are usually very dynamic with a lifetime of 2 to 10 min in most cell types regardless of the substratum^7,11^. MKs podosomes associated to collagen I happened to be very stable. Once established, their lifetime could extent up to 90 min to several hours. Their dynamics was however not inhibited since linear podosomes could also fission back into isolated dot-like podosomes along the fibers before total disassembly (Fig. 3a, lower panel; see Supplementary videos 3–6).

Interestingly, recent data demonstrated that isolated podosomes communicated with their neighbors to elicit a synchronic response to external cues such as matrix stiffness^12,21,25,28^. Podosomes separated by less than 2 µm are inter-connected by dorsal actin filaments, which enable them to form higher-ordered groups (rosettes, belts, clusters). Myosin II also localizes to dorsal actin filaments to promote mechanical coupling^15,27^. We measured the distance separating individual dot-like podosomes aligned along collagen I fibers, prior to linear podosomes formation. Accordingly, 71.08% were separated by less than 1 µm (Fig. 3b,c), the majority around 0.4 to 0.8 µm (Fig. 3d) and fairly evenly spread (Supplementary video 7), perfectly correlating with the model of podosomes interaction reported in other cells^26^.

### MKs podosomes lose Tks adaptators and digestive ability during maturation

Linear invadosomes described in various cell types display a very strong digestive activity linked to the expression of the scaffolding protein Tks5^21,29,30^, together with the presence of MT1-MMP to allow their formation and remodeling function on collagen I^22^. To first assess whether MKs linear podosomes exhibited any matrix-digestive ability, we cultured MKs at different stages of maturation on fluorescent gelatin-coated glass overlayed by collagen I.

**Figure 4:**
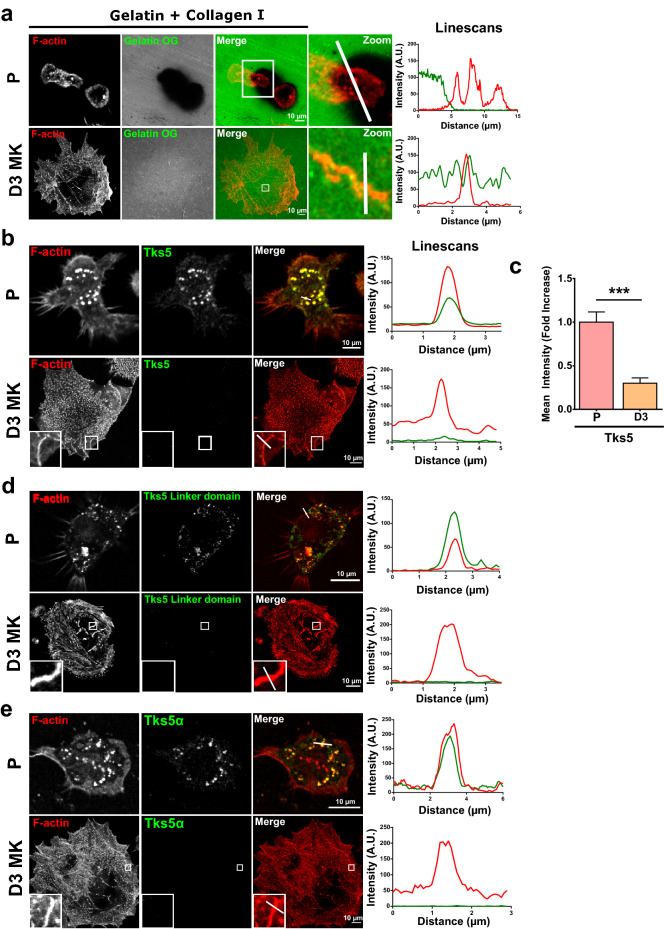
MKs lose their digestive ability together with Tks5 expression during maturation. (**a**) Lin^−^ progenitors (P) and D3MKs were cultured for 6 h on gelatin- Oregon Green 488 (green) containing 100 µg/ml Horm collagen (non labelled) matrix prior to fixation and staining for F-actin (in red). (**b**) P and D3MKs were treated as in (**a**), then stained for F-actin (red) and Tks5 (green). (**c**) Quantification of mean Tks5 intensity per podosome from 3800 podosomes from 18 cells. Values are mean ± s.e.m.***P < 0.001 according to unpaired t-test. (**d**) P and D3MKs were treated as above, then stained for F-actin (red) and Tks5 linker domain (2G6 antibody) or (**e**) Tks5 PX domain (F4 antibody). Linescans show the distribution of fluorescence along the white line. Scale bars = 10 µm. n = number of cells studied.

Lin^−^ progenitors (P) displayed a strong digestive activity, showing degraded areas that spread around the cells, indicating that gelatinases were secreted in the medium (Fig. 4a). This was confirmed by zymography in which supernatants (Spt) and total cell lysates (TL) were analyzed and showed a band of digestion around 90 kDa. Even though the gelatinase activity was stronger in TL, a clear band of digestion was detected in Spt of progenitors, which pointed to a secreted gelatinase, probably MMP9 (only MMP of 90 kDa) (Supplementary Fig. S3b). Accordingly, progenitors podosomes also expressed Tks5α, a hallmark of digestive podosomes or invadopodia, as shown by immunofluorescence using different antibodies (Tks5 and Tks5 linker domain antibodies recognize all Tks5 isoforms, Tks5α is specific for the PX domain (Fig. 4b–e)^31^.

Conversely, neither dot-like nor linear podosomes showed any Tks5 staining in D3MKs (Fig. 4b–e). Westernblotting demonstrated that Tks5 significantly decreased during maturation (Supplementary Fig. S3c). In parallel, no digestive function was associated to linear podosomes in D3MKs as demonstrated by imaging on collagen I-containing fluorescent gelatin and by zymography (Fig. 4a, Supplementary Fig. S3b). D2MKs happened to have already lost degrading capabilities (Supplementary Fig. S3a). It is to note that progenitors expressed Tks4 at podosomes and at the plasma membrane, and its expression was also decreased during MKs maturation (Supplementary Fig. S3d, e).

These data distinguished MKs linear podosomes from linear invadosomes found in other cells, since they do not express Tks5 and do not function as matrix-degradation subcellular domains.

### Mature MKs remodel collagen I matrix through traction of the fibers by linear podosomes

**Figure 5:**
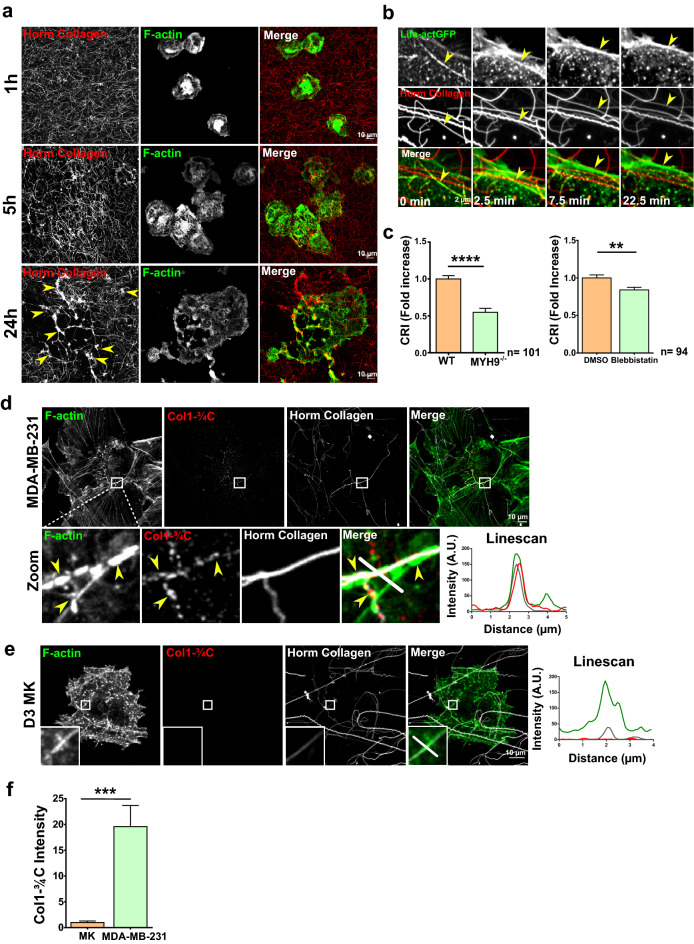
Mature MKs remodel fibrillar collagen matrix through traction by linear podosomes, without collagenase involvement. (**a**) D3MKs were plated on labelled 100 µg/ml Horm Collagen (red), and allowed to spread for the indicated times before fixation and F-actin staining (green). Confocal images were then taken. Scale bars = 10 µm. (**b**) Lifeact-GFP-expressing D3MKs were cultivated on 100 µg/ml labeled-Horm Collagen (red). Confocal videomicroscopy was performed and images taken every 30 s. Yellow arrows show a linear podosome on a collagen fiber. (**c**) Collagen remodeling index (fold increase) was calculated from WT treated or not with DMSO (vehicle) or blebbistatin (20 µM) and MYH9^−/−^ D3MKs cultured on 100 µg/ml of labeled-Horm collagen. (**d**) MDA-MB-231 cells or (**e**) D3MKs were cultured for 6 h on 100 µg/ml of labeled-Horm collagen (white) prior staining for F-actin (green), or collagenase-cleaved collagen type 1 neoepitope (Col1-3/4C, red). Yellow arrows point to a site of collagen cleavage associated to a linear invadosome along fibers bundles. Linescans show the distribution of fluorescence along the white line. Scale bars = 10 µm. (**f**) Graph corresponds to the fluorescence intensity of Col1-^3/4^C in MDA-MB-231 and D3MKs. Values (mean ± S.E.M.) are from 3 to 5 independent experiments. **P < 0.01, ***P < 0.001, ****P < 0.0001 according to the Mann–Whitney test. n = number of cells studied.

MKs maturation seemed to correlate with loss of Tks4/5 and degradative activity. Interestingly, linear podosomes of mature MKs possessed proteins important in mechanosensing and mechanotransduction: (i) their dynamics depended on myosin IIA; (ii) they expressed mechanosensitive proteins such as talin and vinculin which respond to tension by signaling to cellular actin^12,26,32^. Confocal observation of D3MKs confirmed that after 24 h of plating onto collagen I, no degradation was observed (Fig. 5a). Strikingly, the collagen I matrix appeared to have been highly remodeled and followed the shape change of MKs (Fig. 5a) (D4MKs make proplatelets-like protrusions in our experimental setting, as already published^23^). Indeed, already after 5 h and very clearly after 24 h, tangled clumps of collagen I fibers were observed under MKs that attempted to extend protrusions on the loose collagen matrix (Fig. 5a, yellow arrows). This suggested that mature MKs remodeled collagen I by mechanically bending/pulling the matrix fibers, which accumulated under cellular bodies. This property was related to the maturation stage since D2MKs could not form clumps (Supplementary Fig. S4a). To quantify this event, we derived a coefficient, CRI (collagen I remodeling index) that corresponds to the collagen I fibers gathered under the cell relative to MKs areas. Comparison of CRI confirmed that it was a function of maturation, as was the appearance of linear podosomes (Supplementary Fig. S4b).

Confocal videomicroscopy of Lifeact-GFP expressing MKs confirmed that linear podosomes associated to collagen I fibers could develop forces to mobilize them (Fig. 5b, yellow arrow and Supplementary video 8). MYH9^−/−^ MKs or blebbistatin-treated MKs, not only did not form linear podosomes, but were also not able to remodel collagen I matrix, and, therefore, showed a decreased CRI compared to WT MKs (Fig. 5c and Supplementary Fig. S5a,b).

Mechanical remodeling of collagen I fibers was observed in MDA-MB-231 tumoral cells, where MT1-MMP collagenase associated to linear invadopodia helped to increase fibers compliance by selective cleavage, as shown in Fig. 5d using the Col1-^3/4^C antibody (detects collagenase-cleaved collagene I)^22^. Staining of D3MKs with Col1-^3/4^C demonstrated that no collagenolytic activity was associated to MKs linear podosomes (Fig. 5e,f). Accordingly, treatment with 40 µM of GM6001 (broad inhibitor of MMPs) or 100 µM of NSC405020 (specific to MT1-MMP)^33,34^ did not affect linear podosomes formation (Supplementary Fig. S6a) nor function since the remodeling index CRI was not affected (Supplementary Fig. S6b). It is noteworthy that MKs seem to express MT1-MMP in hardly detectable amounts, and not in linear podosomes by opposition to MDA-MB-231 cells (Supplementary Fig. S7a–c).

**Figure 6:**
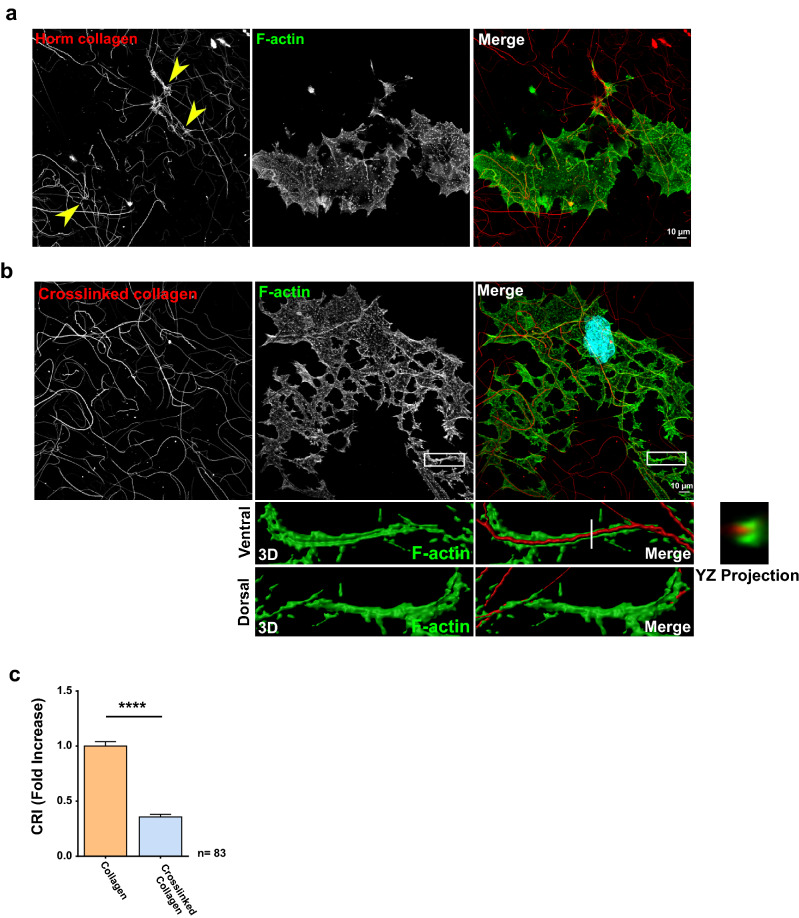
Immobilization of collagen fibers by crosslinking induces MKs to extent protrusions that follow the fibers network. (**a**) Representative image of D3MK cultivated for 24 h on labeled-Horm Collagen (100 µg/ml). Yellow arrows indicate tangled clumps of collagen. (**b**) Representative image of D3MK cultivated for 24 h on crosslinked labeled-Horm Collagen (100 µg/ml). Zooms show 3D surface rendering of a gutter linear podosome surrounding fibers. An orthogonal projection following the axis of the white line is shown (YZ projection). Scale bar = 10 µm. (**c**) Collagen remodeling index (fold increase) of collagen and glutaraldehyde crosslinked collagen. Values (mean ± S.E.M.) are from 4 independent experiments. ****P < 0.0001 according to by the Mann–Whitney test. n = number of cells studied.

To better understand what collagen I fibers traction translated into for D3MKs (they are huge noninvasive cells at this stage) encased in a complex rigid matrix, we crosslinked collagen I fibers with glutaraldehyde prior to allow cells to spread for 24 h. Under these conditions, collagen I could not be moved anymore. The cellular body was then the only deformable entity, leading to the extension of protrusions from MKs that had some similitude to proplatelets, along the stiff collagen I fibers (Fig. 6b compared to 6a). Confocal observation showed that those protrusions bore linear podosomes for most of them (Fig. 6b, orthogonal projection, Supplementary video 9). As expected, the CRI was dramatically reduced (Fig. 6c).

Taken together, these data unravel a new function as traction forces transmitters for linear podosomes in mature MKs, independently of any degradative activity.

### GPVI and ß1 act together as collagen I receptors to drive linear podosomes formation and function

Our data suggested that a strong binding was required between linear podosomes and collagen I. So far, five transmembrane collagen I receptors have been described: the α2ß1 collagen I-binding integrins, the discoidin domain receptors (DDR1 in invadosomes), the Leukocyte-associated immunoglobulinreceptor-1 (LAIR-1), the Glycoprotein VI (GPVI) and the hyaluronan receptor CD44^14,28,35,36^. We did not investigate LAIR-1 because it is specific of leukocytes.

DDR1, a major inducer of linear invadosomes in many cell types, appeared to be weakly expressed in progenitors, but its expression dramatically decreased with maturation (Supplementary Fig. S8a). CD44, another collagen I receptor important in podosomes function and maturation, even though expressed by matured MKs, did not localized in either type of podosomes, but mostly associated to the demarcation membrane system, a membrane reservoir formed by tubular plasma membrane of future proplatelets/platelets (Supplementary Fig. S8b).

**Figure 7:**
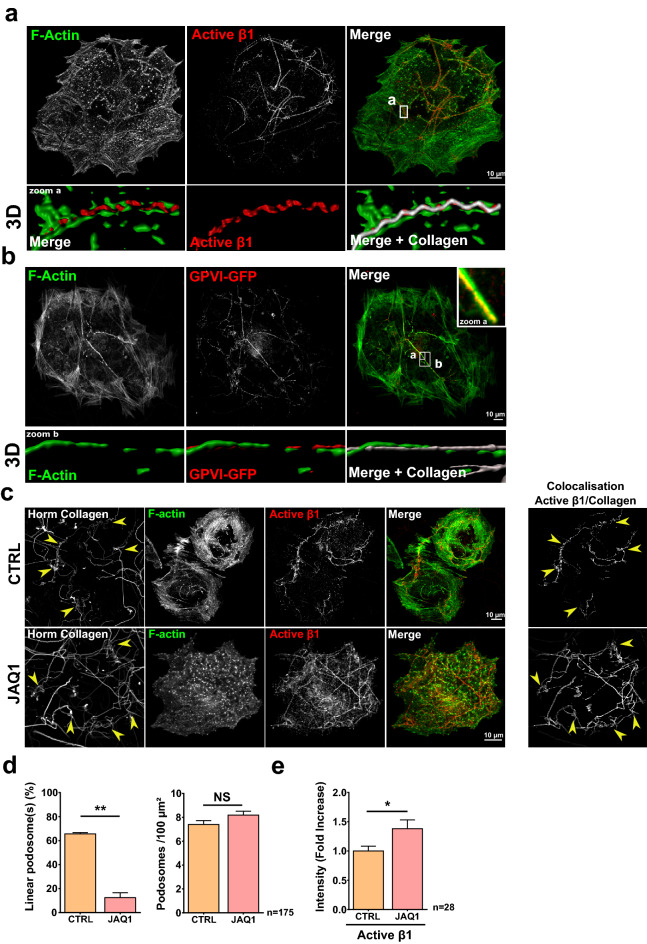
GPVI and the ß1 integrin act as collagen I receptors to generate linear podosomes. (**a**) Representative confocal image of mature MKs cultured for 6 h on 100 µg/ml Horm Collagen, prior to fixation and staining for active ß1 (red) and F-actin (green). 3D corresponds to 3D surface rendering done with the IMARIS software. Scale bar = 10 µm. (**b**) hGPVI-eGFP (red) lentivirus transduced MKs were treated as in (**a**). Zoom a shows a linear podosome above a linear collagen fiber. Zoom b is a 3D surface rendering of the collagen, linear podosome and GPVI-GFP reconstruction using the IMARIS Software. Scale bar = 10 µm. (**c**) CTRL (control) and JAQ1 (GPVI blocking antibody, 10 µg/ml)-pretreated D3MKs were cultured on 100 µg/ml labeled-Horm Collagen, fixed and stained for active ß1 (red) and F-actin (green). Yellow arrows indicate Collagen tangled clumps. Image rendering of the colocalization of active ß1 and Collagen fibers performed as described in “Methods” is shown. Scale bar = 10 µm. (**d**) Quantification of linear podosomes and dot-like podosomes (podosomes) density (for 100 µm^2^) in D3MKs. (**e**) CRI and fluorescence intensity measurements from active ß1 on collagen fibers with or without JAQ1 treatment. Values (mean ± S.E.M.) are from 5 independent experiments. *P < 0.05, **P < 0.01, *NS* not significant according to the Mann–Whitney test. n = number of cells studied.

**Figure 8:**
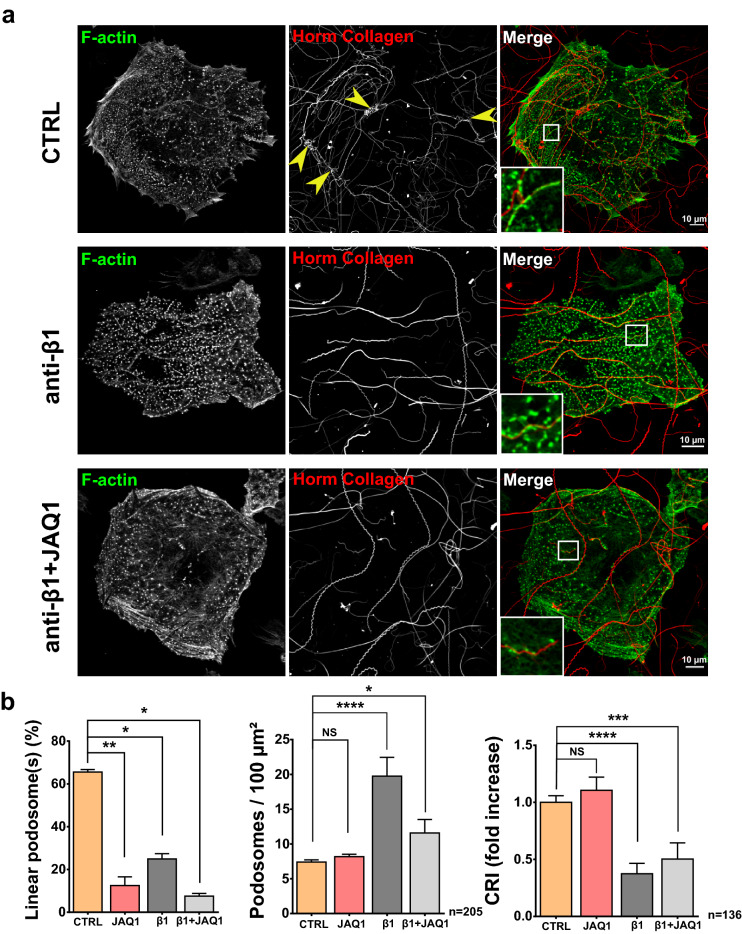
Inactivation of both GPVI and the ß1 integrin dramatically impairs linear podosome formation and traction of collagen I. (**a**) Representative confocal image of mature MKs cultured for 6 h on 100 µg/ml Horm Collagen, prior to fixation and staining for F-actin (green). Control D3MKs (CTRL) and D3MKs pretreated with a blocking ß1-integrin antibody (Purified NA/LE Hamster anti-rat CD29, 10 µg/ml) with or without JAQ1 (GPVI blocking antibody) were fixed and stained for F-actin (green). Yellow arrows indicate Collagen tangled clumps. Scale bar = 10 µm. (**b**) Quantification of linear podosomes or dot-like podosomes (podosomes) density (for 100 µm^2^) and CRI in the indicated cells. Values (mean ± S.E.M.) are from 3 independent experiments. *P < 0.05, **P < 0.01, ***P < 0.001, ****P < 0.0001, *NS* not significant according to the Mann–Whitney test. n = number of cells studied.

The α2ß1 integrin and GPVI are known as the main collagen I receptors in platelets and MKs^37^. Confocal imaging demonstrated that both active-ß1 and GPVI-GFP associated with linear podosomes (Fig. 7a,b). Supplementary videos 10 and 11 showed a strong association between collagen fibers, linear podosomes (F-actin) and the receptors. SIM showed a perfect colocalization of GPVI-GFP, F-actin and cortactin along collagen I fiber (Supplementary Fig. S8c, zoom a and b) bearing linear podosomes. Importantly, inactivation of GPVI with the JAQ1 inhibitor led to a decrease in the percentage of linear podosomes (Fig. 7c,d), suggesting that GPVI is involved in their formation. Surprisingly, the CRI did not change, meaning that another mechanism was partially compensating for GPVI-loss of function (Fig. 8b). Indeed, a crosstalk between GPVI and α2ß1 has been described in platelets and megakaryocytes actin remodeling^38^. Imaging demonstrated a significant increase in active-ß1 when GPVI was inactivated (Fig. 7c,e), which was even more striking when images of the colocalization of active-ß1 and collagen I were extracted (Fig. 7c). Hence, loss of GPVI resulted in hyper activation of the ß1 integrin. We further explored this observation by inactivating ß1 using the blocking 'NA/LE Hamster anti-rat CD29' antibody (referred to as 'anti-ß1' in Fig. 8). Blocking ß1 resulted in a dramatic loss of linear podosomes paralleled by an increase in dot-like podosomes, and strong decrease of collagen I traction as demonstrated by the CRI (Fig. 8a,b). Blocking both GPVI and ß1 did not lead to further changes in the effects (Fig. 8). Altogether, our data demonstrated that both GPVI and ß1 are important for the formation of linear podosomes on collagen I. However, in MKs, ß1 appeared as the prominent receptor to transduce forces required to remodel collagen I, since blocking GPVI had no effect on the CRI, while maximum effect was reached with blocking ß1 (Fig. 8b).

## Discussion

Thrombopoiesis relies on the maturation of small pluripotent progenitors within the bone marrow into giant MKs that will extend PPTs towards medullar sinusoids to finally release platelets in the blood stream. This process is tightly regulated by the interplay of cytokines and contacts with ECM^2,3^. As for other myeloid-derived cells such as dendritic, macrophages or osteoclasts, contacts between MKs and ECM relies on highly dynamics actin-based mechano-adhesive structures called podosomes. They can associate in various type of super-structures in response to the stiffness of the substratum due to their mechanosensing and dynamics properties^9,12,13,17,18^. In bone marrow, despite some discrepancies regarding the matrix composition of the osteoblastic or vascular niches, fibrillar collagen I is the most represented protein^6,39^. However, an uncontrolled accumulation of collagen I fibers, as observed in myelofibrosis, may prevent cells motility contributing to bone marrow ‘suffocation’ and cytopenia. This phenomenon and its repercussions in pathophysiology prompted several groups to study the behavior of maturing myeloid cells in contact with collagen I fibrils, among which MKs^40^.

Few reports detailed MKs interactions with collagen I^7,8^. It was reported that MKs formed dot-like podosomes when spreading onto various matrices such as fibrinogen, fibronectin or basal membranes, on which they displayed degradative capabilities. Alignments along collagen I fibers was mentioned, together with their inability to digest this matrix, which was mostly attributed by the authors to technical difficulties related to the thickness of the fibers^7^. In parallel, the existence of linear invadosomes/invadopodias forming along collagen I fibers and displaying a strong collagenase activity was described in many cell types^21,41^. Herein, we further studied the relationship between podosomes and collagen I fibers during MKs maturation. We found that they assembled and reacted to the stiffness of the fibers by not only aligning along collagen I fibers, but by forming a new linear podosome through fusion or nucleation, and rearrangement of the proteins of MKs dot-like podosomes. These structures were seen after 5 to 6 h of spreading on collagen I, which might explain why they were not observed in other studies that assessed podosomes after only 2 to 3 h of spreading^7^. Importantly, they were totally absent in progenitors, and MKs acquired the ability to assemble them at late stages of in vitro maturation, just prior to PPTs elongation. It is noteworthy that mature MKs directly obtained from the bone marrow also formed linear podosomes (AO and FGI, unpublished data).

Analysis of the molecular composition of both types of podosomes demonstrated that they were quasi-identical: a core composed of F-Actin, cortactin, Arp2/3 and WASp, which was surrounded by talin, vinculin and myosin IIA. However, we observed a striking difference when compared to the progenitors podosomes, which presented high levels of Tks5 associated to a strong digestion ability. Importantly, MKs lost expression of Tks5 in parallel to their loss of degradative potency while maturing. These data once more highlight the importance of Tks5 as a marker of digestive abilities^29,30^. It has been linked to an association with the matrixmetalloproteinase MT1-MMP that was shown to be mandatory for formation and mechanical remodeling of collagen I by MDA-MB-231 tumoral cells^22,42^. Our data clearly demonstrated that MMP activity was not required for MKs linear podosomes to function, and that MT1-MMP was not localized in either dot-like nor linear podosomes in those cells. In our model, modulation of Tks5 expression correlated with a switch of function. Indeed, linear podosomes exerted traction forces on collagen I fibers to move them, leading to ECM remodeling without destroying it, possibly to allow passage of PPTs or large MKs fragment towards the sinusoids. These traction forces elicited by contact with collagen I were observed by formation of collagen clumps under the cells on loose collagen I, while MKs extended protrusions when collagen I was immobilized by crosslinking. This is compatible with the idea that, MKs being giant cells (> 80 µm wide in suspension), making space for them to move as a whole through the ECM to reach the sinusoids would certainly compromise the integrity of both the marrow and vessels.

Importantly, when we compared our data with the composition and function described for the linear invadosomes/invadopodia, we found that major differences in composition accounted for the difference in function i.e. digestion versus traction. As already mentioned, linear podosomes lacked digestive ability, and Tks5 and MT1-MMP were not present in the structure, which is an important difference from other types of invadosome^21,22,29,41^. In line with this observation, one of the factors considered to discriminate podosomes from invadopodia is their lifetime, which is supposed to be from seconds to around 10 min for podosomes, while invadopodia are stable for several hours^42^. Our data demonstrated that in MKs, this factor needs to be reevaluated since we observed that both dot-like and linear podosomes associated to collagen I fibers were stable for 90 min to several hours, despite the fact that they were not displaying any invasive ability. Obviously, new findings make the task of having a clear definition of 'what is what' in the invadosome family a difficult one.

Another conundrum is whether all invadosomes have integrins (mainly ß1 or ß3) as part of the ring. Several studies have demonstrated the presence and even the association of ß1 with MT1-MMP^10,13,14,22,42^. However, major studies on linear invadosomes have demonstrated that in those cases where degradative activity was found, ß1 was either not present in the structure, or that its depletion did not affect the formation nor function of the linear invadosome, leaving space for speculation on the matter^21,22^. However, our study showed that in MKs were linear podosome is essentially a mechanical structure that remodel ECM by transmitting forces without degradation, ß1 is a major player.

Interestingly, we observed two forms of podosomes along collagen I fibers, the linear podosome on thin fibers, and ‘gutter’ linear podosomes on wider fibers, bigger structures likely to transmit more forces to move the big fibers. According to our functional observations and because they shared the same composition, it is likely that they are an adaptation of the same linear podosome to the thickness of the collagen I fiber/bundle.

As already described for the regulation of stress fibers in MKs and platelets over collagen I, GPVI and ß1 were both working together to bind collagen and transduce signal^37^. For years, a widely accepted two steps process identified GPVI as the main primary receptor for collagen I, while in a second time ‘inside-out’ signaling activated ß1 to strengthen signaling. Our data showed that both receptors were required for linear podosome formation, while ß1 was required for traction of collagen I, which makes total sense since it is coupled to mechanosensitive and transducive proteins such as vinculin, paxillin or talin. This supports the actual model, developed after platelets from the GPVI-KO mouse were shown to still being activated by collagen I, stating that both receptors could recognize collagen I and work in parallel for a better response^38,43^.

We recently published that in vivo, MKs used a transcellular mechanism that involved podosomes at the extremity of PPTs or protrusions in contact with sinusoids endothelium to cross into the blood stream at sites devoid of ECM^16^. Since MKs were shown to degrade fibrinogen (enriched in sinusoids vicinity) and basal membrane, a likely hypothesis is that collagen I fibers are used for guidance towards the sinusoids. Active digestion might then be retained or reactivated at the extremity of the protrusion upon contact with the endothelium or vascular niche. Another more provocative possibility stems from a report^4^ that relates that MKs do not migrate from the osteoblastic niche towards the vascular niche during differentiation, but all differentiate within 5 µm of the vessels. According to our data, one could imagine that progenitors at very close range of the endothelial barrier would digest the ECM, thereby creating space for MKs podosomes to sense weaker spots and extend protrusions towards endothelial cells using linear podosomes and guidance of collagen I fibers.

In conclusion, we demonstrated for the first time in primary cells that differentiation of progenitors into mature cells, here MKs, led to a switch in composition, structure and function of podosomes, which adapted to the constrained microenvironment (ECM and cytokines) to accommodate the changes required for mature cell function. We found that podosomes, known to be able to reorganized in super-structures by forming strongly packed clusters, could also reorganize their proteins scaffold to create structures encompassing the same molecules in a rearranged order to fullfil their new function. Here, the core of the dot podosome became a line over collagen, while receptors and linkers from the ring aligned along the core and fiber. These findings support the concept that various members of the invadosome family could represent different stages of maturation of invadosomes according to the ECM composition and stiffness, or the specificity of the cell type that express them.

## Methods

### Reagents and antibodies

Stempro medium used for MK cultures was from Invitrogen. mSCF (murin SCF) and mTPO (murin TPO) were from Peprotech, France. Fibrinogen was from Sigma Aldrich, France. Horm collagen was purchase from Takeda, Austria. Alexa Fluor™ 647 NHS Ester (A37573) were obtained from Invitrogen. Gelatin-Oregon Green 488 conjugate (G-13186) was from Life Technologies. NSC405020 (4902) was from Tocris Bioscience. GM6001 (364205) was from Calbiochem. Blebbistatin (B0560) was from Sigma Aldrich. The following primary antibodies were used: cortactin (clone 4F11, Millipore), myosin IIA (Sigma Aldrich), JAQ1 (EmfretAnalytics). DAPI (1050, Euromedex), Alexa Fluor 633- 594- 488-conjugated phalloidin (A22284, A12381, A12379, Invitrogen), anti-active β1 (9EG7 BD Biosciences), DDR1 (5583T Ozyme), Tks5 (sc376211 Santa Cruz), Tks4 (09-260, Sigma Aldrich), HSC70 (sc59560 Santa Cruz), Talin (8d4, Sigma Aldrich), Arp2/3 complex subunit 2 (07-227, Sigma Aldrich), Vinculin (hVIN-1, V9264 Sigma Aldrich), CD44 (217594, Millipore), Col1-3/4C (0217-050, Immunoglobe), MT1-MMP (MAB3328, Millipore), β1- blocking antibody ('NA/LE Hamster anti-rat CD29' BD Biosciences, 555002). The following secondary antibodies were used: Anti-Mouse IgG (H + L) HRP Conjugate (W4021, Promega), Anti-Rabbit IgG (H + L) HRP Conjugate (W4011, Promega). Goat anti-Rat IgG (H + L)-Alexa Fluor 555 (A21434), Goat anti-Mouse IgG (H + L)- Alexa Fluor 555 (A21422), Goat anti-Rat IgG (H + L)-Alexa Fluor 647 (A21247), Goat anti-Mouse IgG (H + L)-Alexa Fluor 647 (A21235), Goat anti-Rabbit IgG (H + L)-Alexa Fluor 555 (A21428), F4 rabbit monoclonal (against Tks5α PX domain) and 2G6 mouse monoclonal (against Tks5 linker domain) were already described^31^.

### Animals

#### Mice

C57BL/6 background mice were housed in the Anexplo (http://anexplo.genotoul.fr) vivarium according to institutional guidelines. Mice were housed in a 12 h light/dark cycle, in a humidity and temperature (22 ± 2 °C)-controlled environment with ad libitum access to food and water. All experiments were performed on 8–12-week-old mice (male and female). Ethical approval for animal experiments was obtained from the French Ministry of Research in compliance with the ARRIVE guidelines (https://arriveguidelines.org) and in agreement with European Union guidelines (APAFIS#3627-20l6011516566853 v4).

All experimental animals were anesthetized by using Rompun 2% (Bayer, France) and Zoletil 100 (Virbac, France) in equal volumes prior to euthanasia via cervical dislocation. The floxed Myh9 strain was crossed with the platelet factor 4 (PF4)-Cre mice as described previously to obtain animals with deletion of exon 1 of the Myh9 gene (Myh9^−/−^ mice) in the megakaryocytic lineage^44^.

#### In vitro megakaryocytes differentiation and cell culture

BM cells were obtained from femur and tibia of C57BL/6 mice by flushing, and lineage depletion (Lin^−^) was performed using antibodies to CD16/CD32+ (2.4G2, 553142, BD), Gr1+, B220+ (RA3-6B2, 553086, BD) and CD11b+ (M1/70, 13-0112-85, Invitrogen). The remaining cell population was cultured in 2.6% nutrient-supplemented Stempro medium with 2 mM l-glutamine, 100 IU/ml penicillin, 50 mg/ml streptomycin, and 20 ng/ml mSCF and 50 ng/ml of mTPO for 3 days. Enrichment in mature MKs was done on a BSA gradient.

MDA-MB-231 were obtained from ATCC. MDA-MB-231 were grown in DMEM medium supplemented with 10% SVF. In both cases, medium was supplemented with 100 IU/ml penicillin and 50 mg/ml streptomycin (In vitrogen). Cells were cultured at 37 °C under 5% CO_2_. When inhibitors were used, cells were pre-treated for 30 min with the indicated doses prior to being platted.

### Lentivirus production and transduction

Transduction was performed on Lin^−^ population at an MOI of 1 for 1 day, then 50 ng/ml mTPO were added for 3 additional days. hGPVI-eGFP noted as GPVI-GFP was given by S.P.Watson, University of Birmingham, England)^45^. The encoding sequence was amplified by PCR and cloned in frame into the N174-MCS (Puro) vector (Addgene #81068) linearized with Not1 and the In-fusion kit (Takarta Bio) according to the manufacturer’s instructions. Primers used:

Forward 5′-TTCTTCGAAGCGGCCGCATGTCTCCATCCCCGACCG-3′ and Reverse: 5′-ACAATGCATGCGGCCGCTTACTTGTACAGCTCGTCCATGCC-3′.

GFP-WASp-encoding plasmid was a gift from Adrian Thrasher (University College London). Lifeact-EGFP lentiviral construct was already described^46^.

### Gelatin degradation assay

Coverslips were coated with Oregon green gelatin, fixed with 0.5% glutaraldehyde for 20 min at RT. After washing three times with PBS, Horm collagen has been added (100 µg/ml) and polymerized for 1 h at 37 °C and washed 3 times with PBS. Cells were seeded on coated coverslips and incubated 6 h before fixation and staining.

### Western blotting

Proteins were extracted with 2× Laemmli buffer and analyzed by SDS-PAGE and Western blotting. Immunoreactive bands were detected by chemiluminescence using chemidoc (Bio-Rad Laboratories, Marnes la Coquette, France) with the SuperSignal detection system (Pierce Biotechnology Inc., Rochefield, IL, USA).

### Zymography

Lin^−^ cells were cultured for at least 3 days with SCF (20 ng/ml) and mTPO (50 ng/ml), separated to matures MKs with BSA gradient, remaining Lin^−^ and matures MKs have been cultured once again for 6 h in separated wells with new STEMPRO medium. Abcam Gelatin Zymography protocol has been used and tested for MKs supernatants and lysis, Lin^−^ supernatants and lysis. Same samples have been tested in Western Blot with HSC70 as loading control.

### Horm collagen labelling and coating

To visualize the collagen network, we labeled 1 mg/ml Horm collagen with 10 µg/ml Alexa Fluor 647 carboxylic acid succinimidyl ester for 5 min at room temperature. Collagen polymerization was then induced by adjusting collagen concentration to 100 µg/ml according to the manufacturer’s instructions, adding some dilution to imaging chamber or slides*. *Polymerization was allowed for 1 h at 37 °C, and 3 PBS washes were performed before use.

### Imaging

For immunofluorescence, MKs were allowed to spread onto fibrinogen (100 µg/ml) or Horm collagen (100 µg/ml) coated dishes for the indicated time. Crosslinking of fibrillar collagen I was achieved by 1 h incubation in 0.5% Glutaraldehyde in PBS. 5 PBS washes were then performed before exposing the coated slides under light for 2 h to bleach autofluorescence before use. To block GPVI, we pre-treated MKs with 0.5 µg/ml JAQ1 for 30 min prior seeding. Cells were fixed and permeabilized in one step for 30 min in PBS (phosphate buffered saline) with 3.7% formaldehyde and 0.05% Triton X-100. Samples were saturated in 3% fatty acid free BSA in PBS. Incubation with primary antibodies, fluorescent secondary antibodies, AlexaFluor-labeled phalloidin or DAPI was performed for 30 min at room temperature. Confocal images were captured with a LSM780 operated with Zen software using a 63×, 1.4 NA Plan Apochromatic objective lens (Carl Zeiss). Linescans and 3D surface rendering were done respectively with Fiji and the Imaris 8.2 software (Bitplane AG, Zurich, Switzerland). Structure illumination microscopy (SIM) was performed with an ELYRA.PS1 (Carl Zeiss), using a 63×, 1.4 NA Plan-Apochromat lens and three rotations of a 51-lm grid. Images were captured with a sCMOS camera (Hamamatsu) 63× using oil immersion lens (Carl Zeiss).

Image processing to obtain the colocalization image of active ß1 and collagen was realized with Fiji. Briefly, a binary mask (0 for background and 1 for the collagen I network) was generated from the channel showing labelled-collagen. Then, the mask was applied on the image corresponding to the active ß1 channel of the same cell. The resulting image corresponded to only active ß1 signal that colocalized with collagen I (Fig. 7c). The ‘Collagen Remodeling Index’ or CRI is a standardized method to quantify the amount of collagen accumulated through remodeling in a fixed MK area. Using Fiji, we generated a binary mask on the channel showing the MKs. This allowed to (i) get the area of the cell; (ii) eliminate the signal outside the mask. The mask was then applied to the collagen channel, the amount of fluorescent collagen within the mask assessed. Then, numbers were standardized to a MK area of 100 µm^2^.

### Statistical analyses

Data are expressed as mean ± SEM. For group comparisons, data were tested for Gaussian distribution. Then, a Student t-test (Gaussian) or Mann–Whitney U test (non-Gaussian) were used to compare individual groups; multiple groups were compared by Kruskall–Wallis tests, with Dunn’s post-hoc test. Statistics were performed using Graphpad Prism 6.0. *P < 0.05; **P < 0.01; ***P < 0.001; ****P < 0.0001.

## Supplementary Information

Supplementary Figures.

Supplementary Video 1.

Supplementary Video 2.

Supplementary Video 3.

Supplementary Video 4.

Supplementary Video 5.

Supplementary Video 6.

Supplementary Video 7.

Supplementary Video 8.

Supplementary Video 9.

Supplementary Video 10.

Supplementary Video 11.
