# Supplementary material for: Megakaryocytes form linear podosomes devoid of digestive properties to remodel medullar matrix
Source: Sci Rep. 2022 Apr 15;12:6255. doi: 10.1038/s41598-022-10215-x (PMC9012751; doi:10.1038/s41598-022-10215-x)
Supplement: Supplementary file 1 — Supplementary Figures. [file 41598_2022_10215_MOESM1_ESM.pdf]

Supplementary informations

Megakaryocytes form linear podosomes devoid of digestive properties to remodel medullar matrix.

Antoine Oprescu[1], Déborah Michel[1], Adrien Antkowiak[1], Elodie Vega[1], Julien Viaud[1], Sara A Courtneidge[2], Anita Eckly[3], Henri de la Salle[3], Gaëtan Chicanne[1], Catherine Léon[3], Bernard Payrastre[1,4]& Frédérique Gaits-Iacovoni[1,5]*

[1]INSERM, UMR1297, Université Toulouse III, Institut des Maladies Métaboliques et Cardiovasculaires, Toulouse, France

[2]Oregon Health & Science University, Department of cell, development and cancer biology, Oregon, USA

[3]INSERM, UMRS949, Université de Strasbourg, Etablissement Français du Sang-Alsace, Strasbourg, France

[4]CHU de Toulouse, laboratoire d'Hématologie, Toulouse, France

[5]Molecular, Cellular and Developmental Biology Department (MCD, UMR5077), Centre de Biologie Intégrative (CBI, FR3743), University of Toulouse, CNRS, UPS, 31062 Toulouse, France

*Correspondence to: frgaits@gmail.com

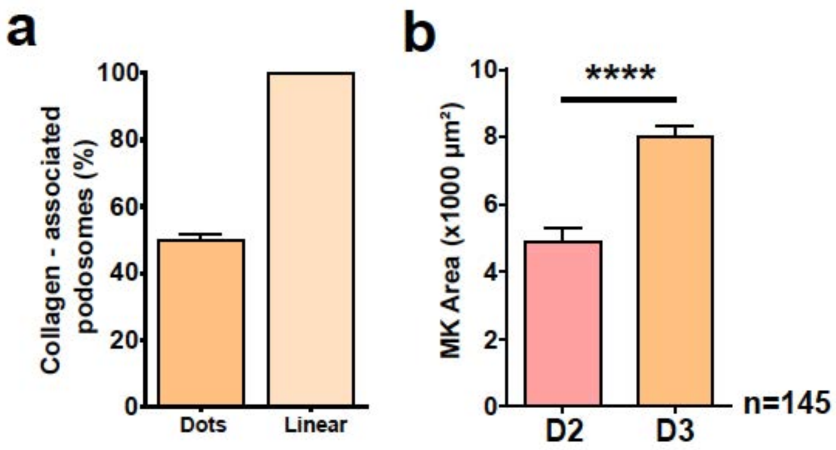

Supplementary Figure S1. Supplementary parameters of mature MKs.

(**a**) Quantification of dot podosomes and linear F-actin structure in contact with collagen (% of linear). (**b**) Comparison of D2 and D3MKs Area. Values (mean ± s.e.m.) are from 4 independent experiments. ****P<0.0001 according to the Mann-Whitney test.

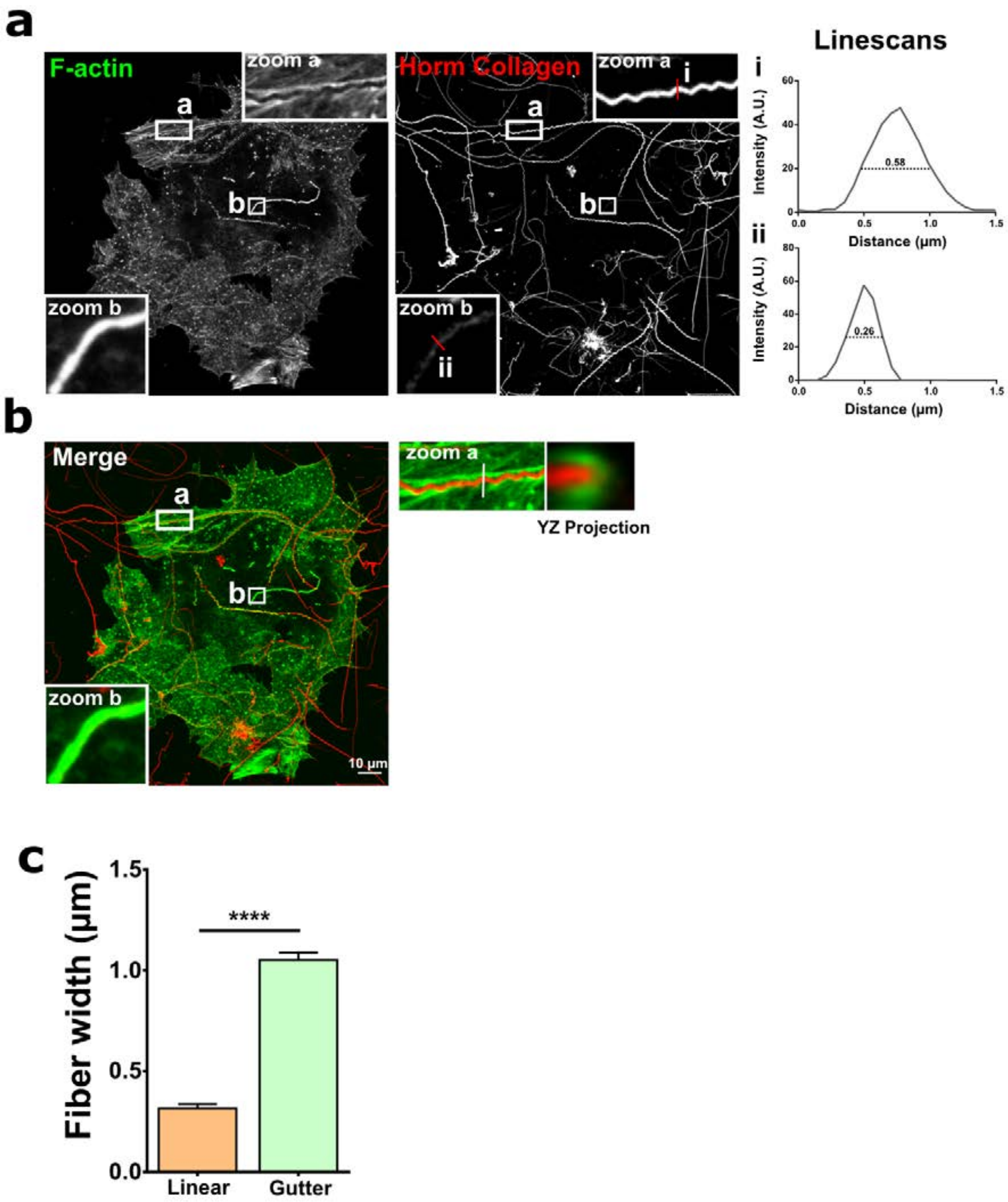

Supplementary Figure S2. Analysis of the width of the collagen fibers in relationship with the type of linear podosome generated.

(**a**) Representative images of D3MK cultured for 5 hours on 100μg/ml labeled-Horm Collagen (red), fixed and stained for F-actin (green). Scale bar = 10μm. Zoom a of a thick fiber shows a gutter podosome and zoom b a thin linear podosome. Linescans show the width of the collagen fibers supporting the gutter (i) and linear podosome (ii). (**b**) YZ projection shows the F-actin gutter surrounding a collagen fiber. (**c**) Comparison of collagen fiber width supporting linear podosomes or the gutter. Data are shown as mean ± s.e.m. ****P<0.0001, according to unpaired t-test.

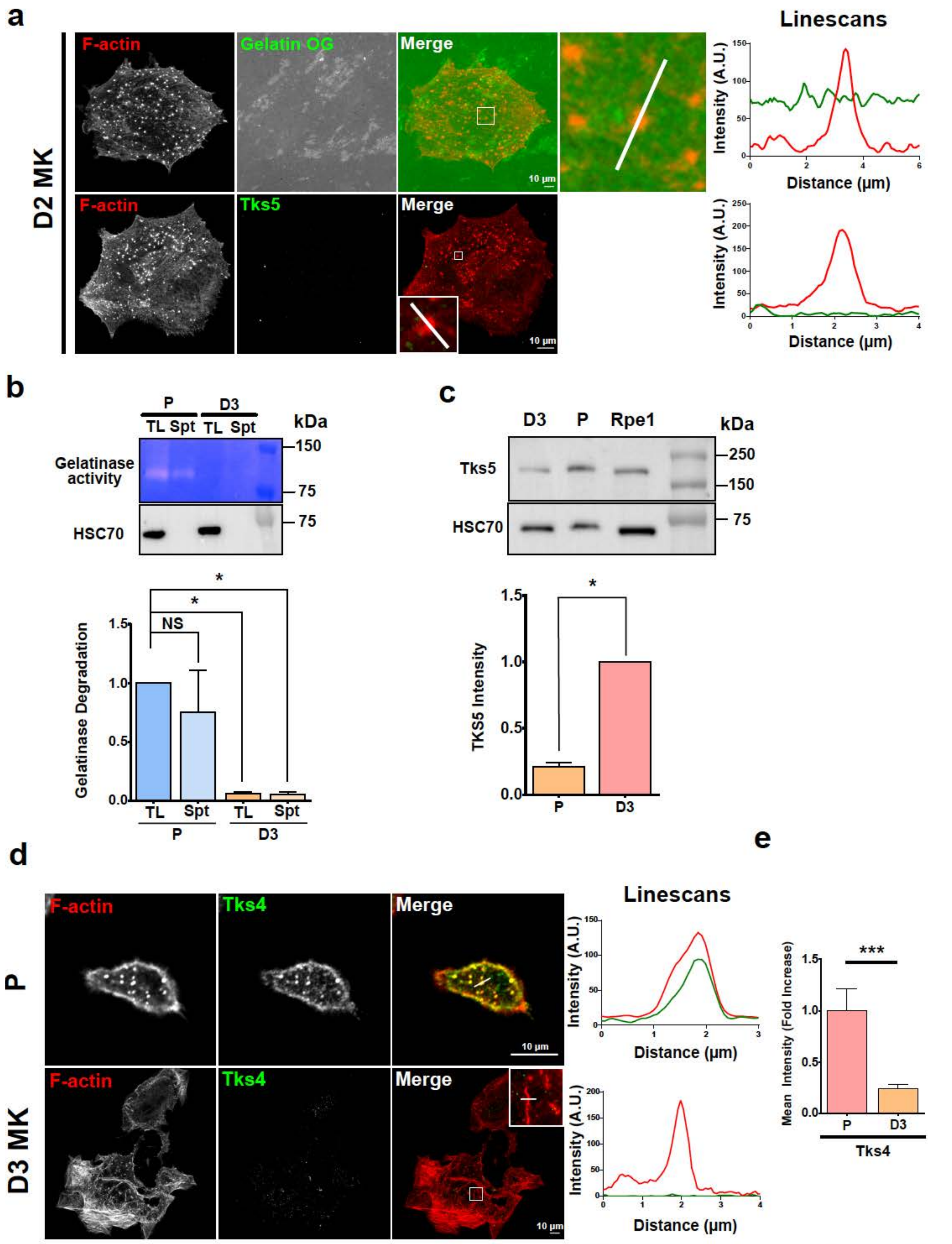

Supplementary Figure S3. Differentiating MKs lose digestive ability and Tks expression. (**a**) D2 and D3MKs were cultured for 6 hours on gelatin- Oregon green 488 (green) containing 100 μg/ml Horm collagen (non labelled) matrix and then fixed and permeabilized

before staining for F-actin (red). Linescans show the distribution of fluorescence along the white line. Scale bars = 10 μm. (**b**) Zymography was performed on Supernatants (Spt) and total lysates (TL) from D3MKs (D3) and Lin- Progenitors (P). Lysates were run on a gel containing gelatine substrate, digestion was evidenced by white bands where gelatin has been digested over the blue background of coomassie staining of the gel. Western Blotting assessing the level of HSC70 was used as loading control. The graph represents a quantification by densitometry of three independent experiments. (**c**) Lysates from D3MKs (D3) or Lin- Progenitors (P) were subjected to Western blotting to measure levels of Tks5. Lysate from Rpe1 cells was used as positive control. HSC70 was used as control loading. The graph represents a quantification by densitometry of three independent experiments. (**d**) P and D3 MKs were cultured for 6 hours on gelatin- Oregon green 488 (green) containing 100 μg/ml Horm collagen (non labelled), fixed, and stained for F-actin (red) and Tks4 (green). Linescans show the distribution of fluorescence along the white line. Scale bar = 10 μm. (**e**) Quantification of mean Tks4 intensity per podosome from at least 491 podosomes of 8 P and 8 D3. Values are presented as mean ± s.e.m. *$P<0.05$, ***$P<0.001$ according to the Mann-Whitney test.

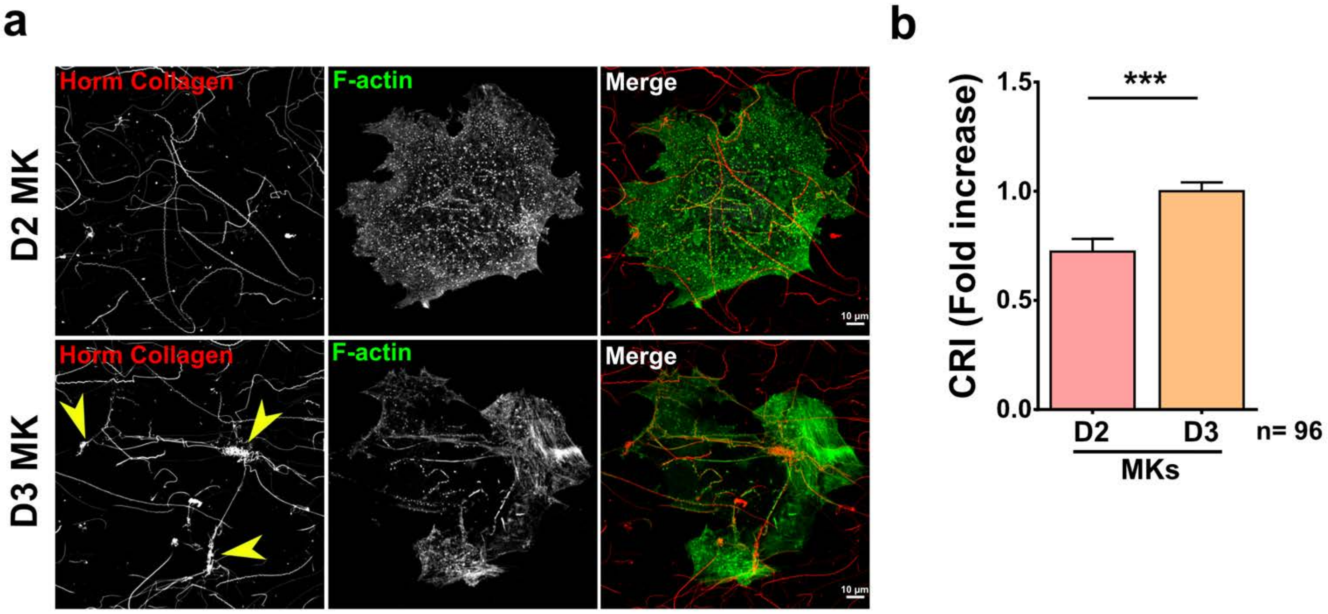

Supplementary Figure S4. Collagen traction ability appears when mature MKs form linear podosomes.

(**a**) Representative images of D2 and D3MKs cultured for 5 hours on 100μg/ml of labeled-Horm Collagen (red), prior to processing and F-actin staining (green). Yellow arrows indicate Collagen tangled clumps. Scale bar = 10μm. (**b**) Quantification of CRI fold increase between D2 and D3MKs. Data (mean ± s.e.m.) are from 4 independent experiments. ***P<0.001, according to the Mann–Whitney test.

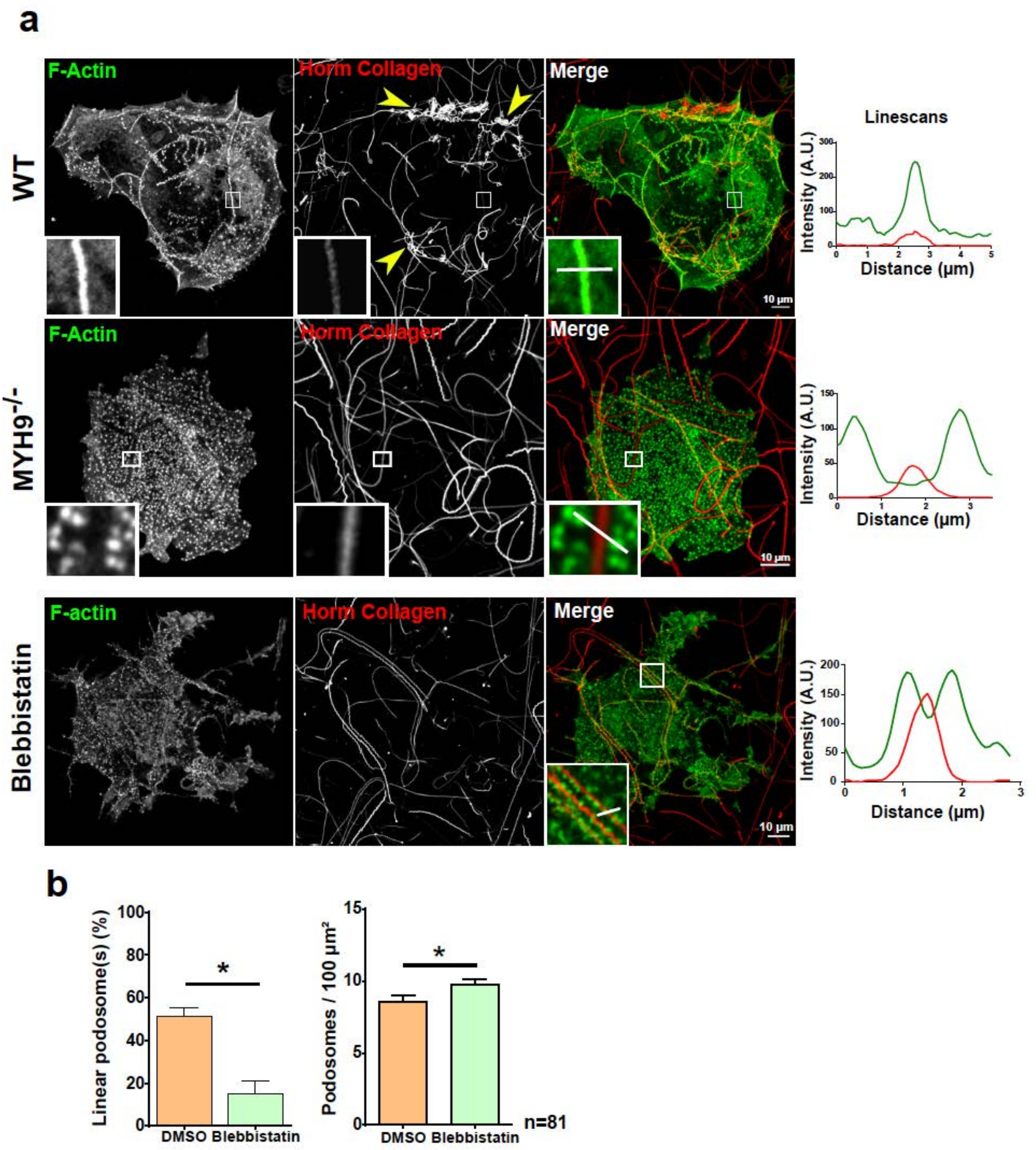

Supplementary Figure S5. Myosin II function is required to form linear podosomes on collagen fibers.

(**a**)WT treated or not with DMSO (vehicle) or blebbistatin (20 µM) and MYH9$^{-/-}$ D3MKs were cultured on 100 µg/ml of labeled-Horm collagen (red) prior to F-actin staining. Yellow arrows indicate Collagen tangled clumps. Linescans show the distribution of fluorescence along the white line. Scale bars = 10 µm. (**b**) Quantification of percentage of WT (DMSO) and blebbistatin-treated MKs forming linear podosomes or podosomes density (for 100 µm^2). Values (mean ± s.e.m.) are from 5 independent experiments. *P<0.05 according to the Mann-Whitney test. n = number of cells studied.

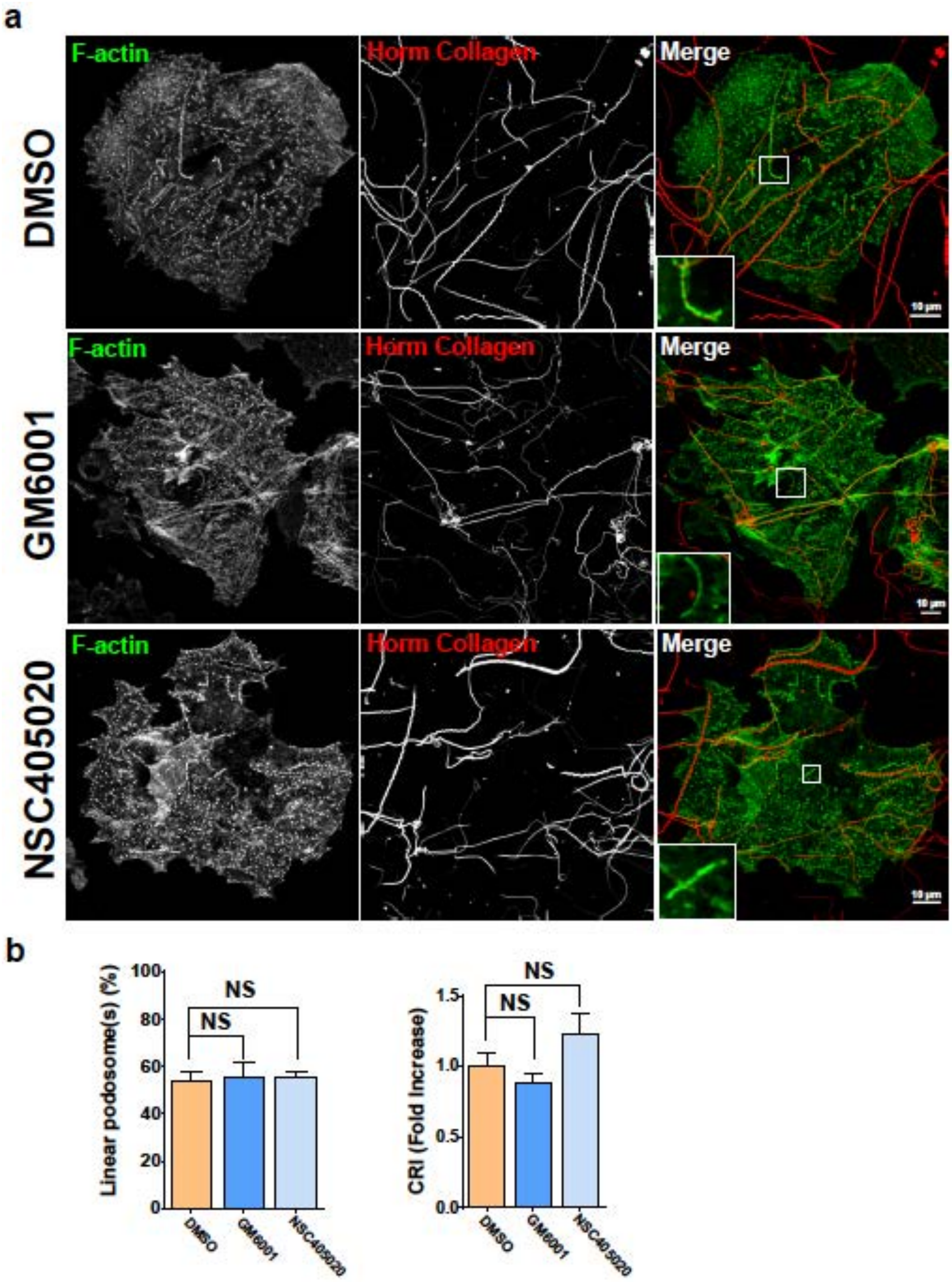

Supplementary Figure S6. MMP activity is not require for linear podosomes formation nor collagen traction ability

(**a**)WT D3MKs treated by either DMSO (vehicle) or GM6001 (broad spectrum MMP inhibitor-40 μM) or NSC405020 (MT1-MMP specific inhibitor-100 μM) were cultured on 100 μg/ml of labeled-Horm collagen (red) prior to F-actin staining. Scale bars = 10 μm. (**b**) Quantification of percentage of MKs forming linear podosomes or podosomes density (for 100 μm^2). Values (mean ± s.e.m.) are from 3 independent experiments. NS refers to not significant according to the Mann-Whitney test (linear podosomes) and the unpaired *t* test (CRI).

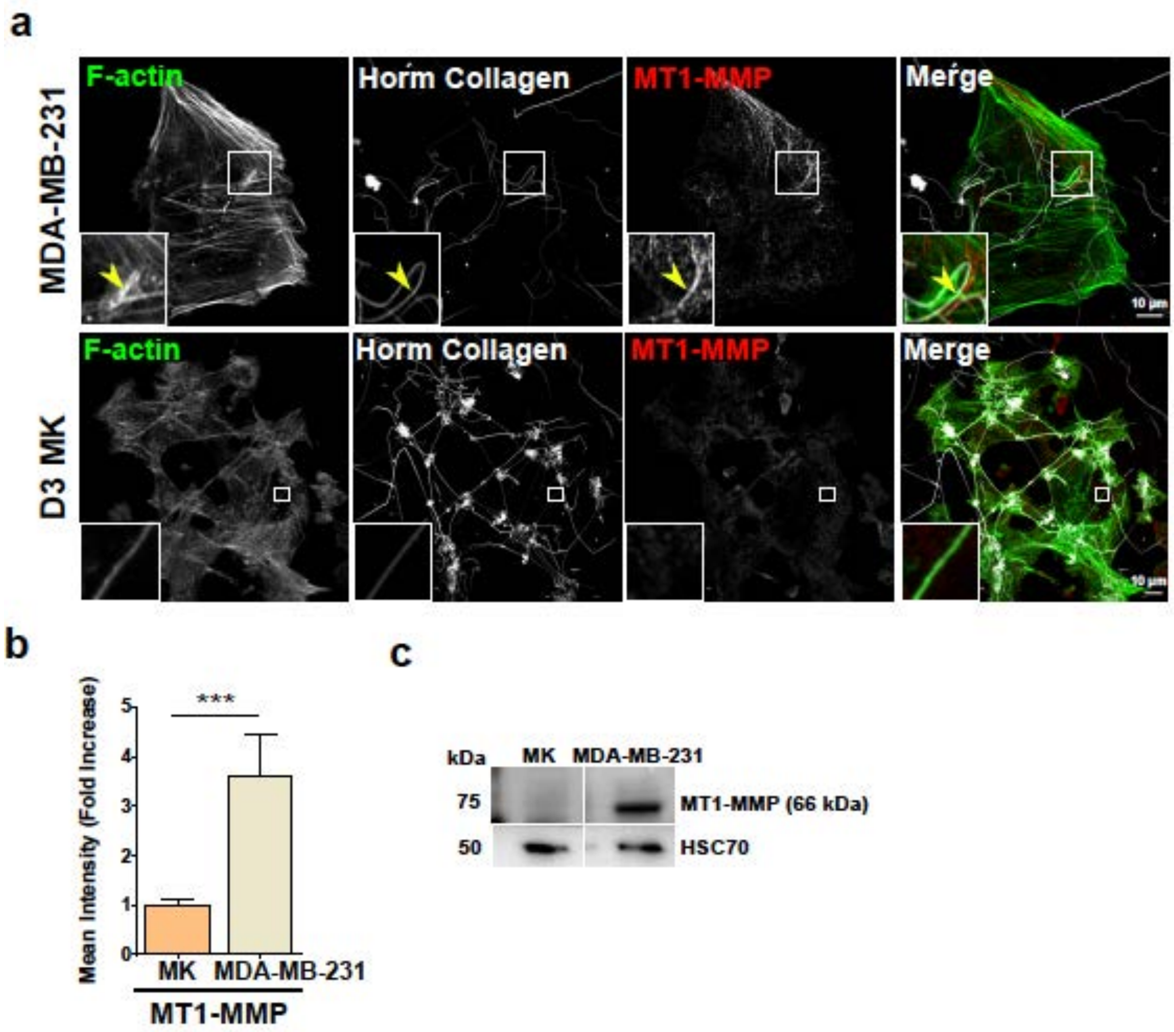

Supplementary Figure S7. MT1-MMP is not part of MK linear podosomes.

(**a**) Representative images of MDA-MD-231 tumoral cells and D3MKs cultured on 100μg/ml of labeled Horm Collagen for 5 hours, were processed and stained for F-actin (green) and MT1-MMP (red), prior to confocal imaging. Scale bar = 10μm. (**b**) Graph represents the mean fluorescence intensity corresponding to MT1-MMP staining. Values (mean ± s.e.m.) are from 3 independent experiments. ***P<0.001, according to the Mann–Whitney test. (**c**) Lysates from D3MKs (MK) or MDA-MB-231 cells were subjected to Western blotting with antibodies against MMP9 and MT1-MMP. HSC70 was used as loading control.

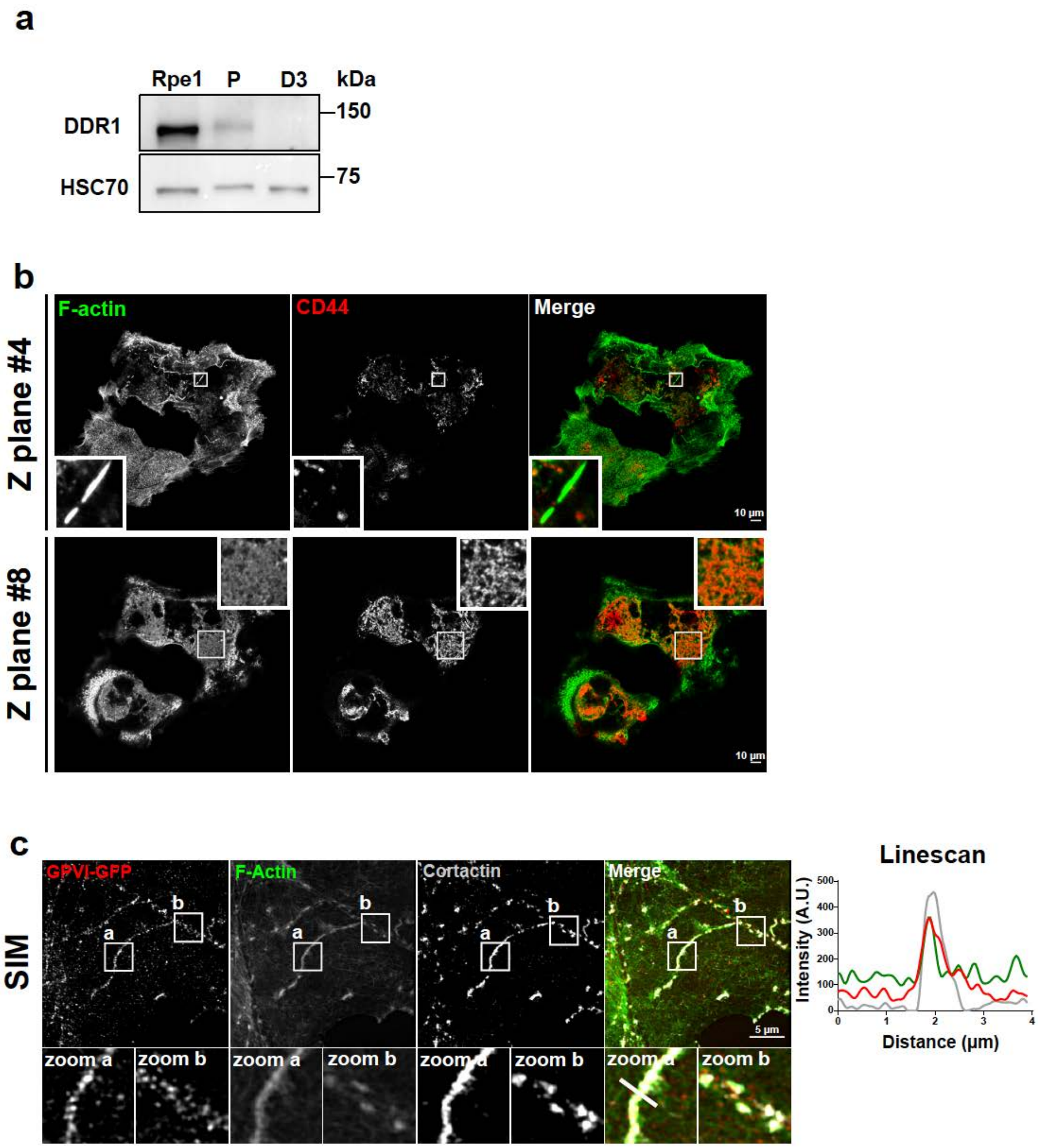

Supplementary Figure S8. Analysis of putative collagen I receptors in mature MKs.

(**a**) Lysates from D3MKs (D3) or Lin- Progenitors (P) were subjected to Western blotting. Anti-DDR1 assessed the level of this receptor. Lysate from Rpe1 cells was used as positive control. HSC70 was used as loading control. (**b**) Representative images of D3MKs cultured on 100μg/ml of un-labeled Horm Collagen for 5 hours, were processed and stained for F-actin (green) and CD44 (red). Two Z sections of the same confocal stack are shown: Z plane #4 corresponds of the ventral surface of the cell, were podosomes can be found; Z plane #8 is taken in the middle of the MK, where the typical tubular complex demarcation membrane

system can be observed. Scale bar = 10μm. (**c**) Transduced GPVI-GFP (red) D3MKs were cultured on 100μg/ml Horm Collagen for 5 hours, prior to staining for F-actin (green) and Cortactin (gray). SIM imaging was then performed. Linescans show the distribution of fluorescence along the white line. Scale bar= 5μm.

Supplementary Figure S3b

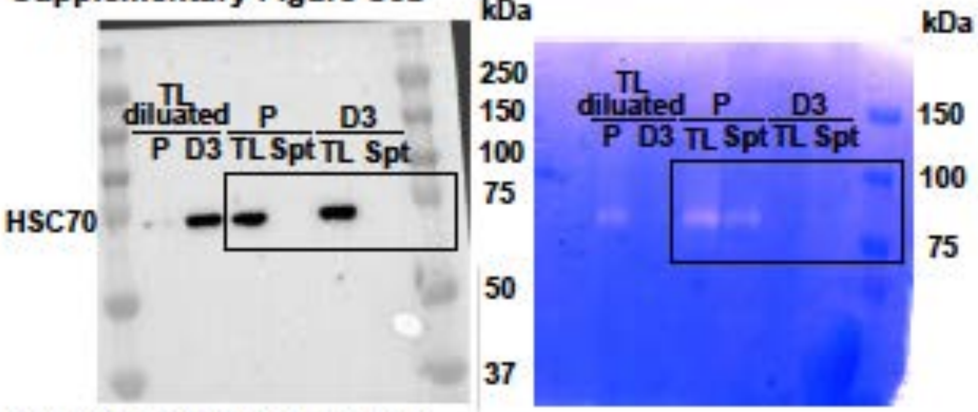

Supplementary Figure S3c

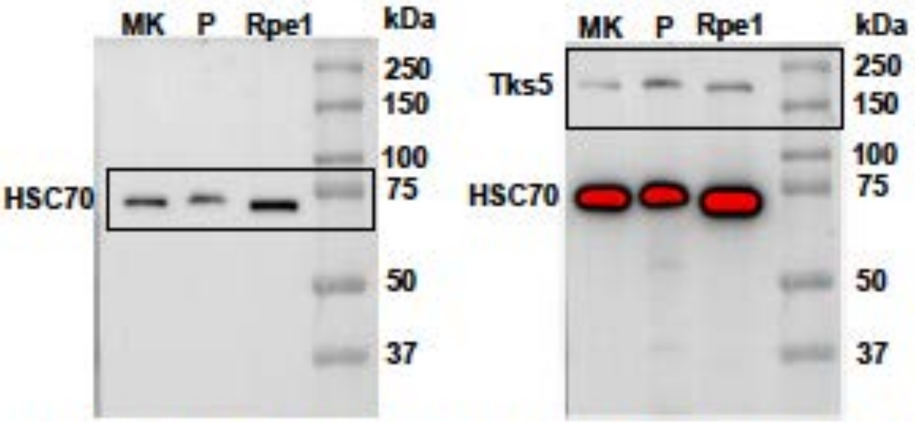

The membrane was first probed with anti-HSC70 antibodies (left membrane), then reprobed without stripping with anti-Tks5 antibodies (right membrane). Longer exposure was required to see the high molecular weight Tks5.

Supplementary Figure S8a

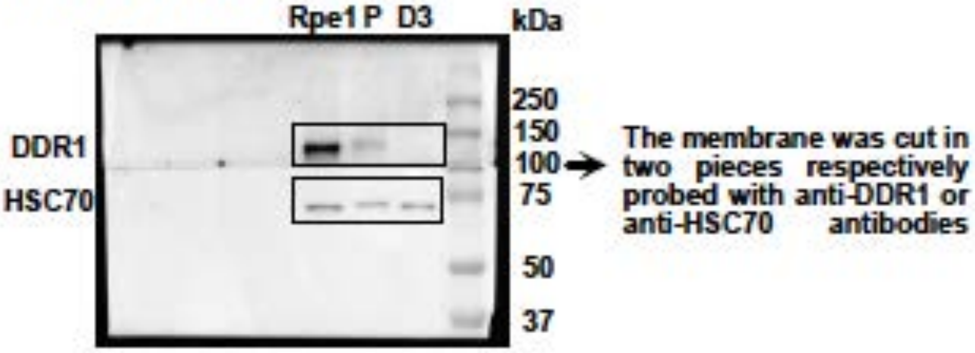

The membrane was cut in two pieces respectively probed with anti-DDR1 or anti-HSC70 antibodies

Supplementary Figure S7c

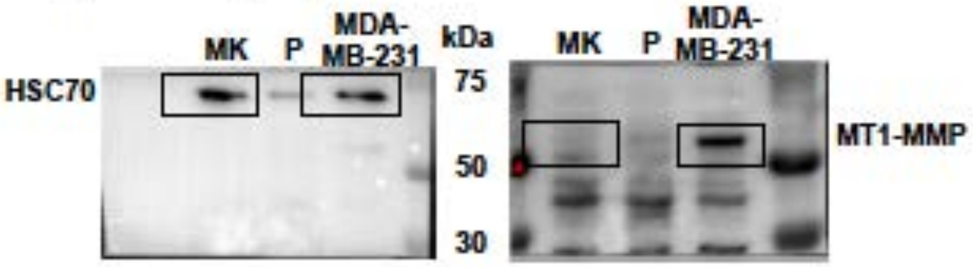

Supplementary Figure S9. Uncropped images of immunoblots relative to Supplementary Figure S3, S7 and S8.

Supplementary Video 1

Rotating view of 3D surface rendering collagen fiber (red) with classical dot podosomes (green) aligned along the fiber in D2MKs.

Supplementary Video 2

Rotating view of 3D surface rendering collagen fiber (red) with linear F-actin structure (green) over the fiber in D3MKs.

Supplementary Video 3

Confocal videomicroscopy of dynamic podosomes fusion (visualized by Lifeact-GFP) in D3 MKs on Horm Collagen. 1 frame every 9.8 seconds.

Supplementary Video 4

Confocal videomicroscopy of dynamic small linear podosomes fusion (visualized by Lifeact-GFP) in D3MKs on Horm Collagen. 1 frame every 20 seconds.

Supplementary Video 5

Confocal videomicroscopy showing linear podosomes formation by nucleation from dot podosomes, then fusion (visualized by Lifeact-GFP) in D3MKs on Horm Collagen. 1 frame every 43.9 seconds.

Supplementary Video 6

Confocal videomicroscopy showing linear podosomes fission that reverted to dot podosomes, highlighting the high dynamics of these structures (visualized by Lifeact-GFP) in D3MKs on Horm Collagen. 1 frame every 13.9 seconds.

Supplementary Video 7

Rotating view of 3D surface rendering collagen fiber (red) with evenly spread dot podosomes (green) aligned along the fiber in D3MKs.

Supplementary Video 8

Confocal videomicroscopy showing a linear podosome (green) moving a thin collagen fiber (red) in Lifeact-GFP Transduced D3MKs on labeled-Horm Collagen. 1 frame every 29.8 seconds.

Supplementary Video 9

Rotating view of 3D surface rendering crosslinked collagen fiber (red) with a gutter-like linear podosome (green) in D4MKs (D3MKs cultured 24h on crosslinked collagen).

Supplementary Video 10

Rotating view of 3D surface rendering collagen fiber (grey), active ß1 integrin (red) and F-actin (green) in D3MKs.

Supplementary Video 11.

Rotating view of 3D surface rendering collagen fiber (grey), GPVI-GFP (red) and F-actin (green) in D3MKs.
